# Limitations of cardiothoracic ratio derived from chest radiographs to predict real heart size: comparison with magnetic resonance imaging

**DOI:** 10.1186/s13244-021-01097-0

**Published:** 2021-11-03

**Authors:** Paulius Simkus, Manuel Gutierrez Gimeno, Audra Banisauskaite, Jurate Noreikaite, David McCreavy, Diana Penha, Monika Arzanauskaite

**Affiliations:** 1 Radiology Department, Liverpool Heart and Chest Hospital, Thomas Drive, Liverpool, L14 3PE UK; 2 Department of Radiology, Lithuanian University of Health Sciences, Eiveniu 2, 50161 Kaunas, Lithuania; 3 Cardiovascular Research Center-ICCC, Hospital de La Santa Creu I Sant Pau, IIB-Sant Pau, Barcelona, Spain

**Keywords:** Cardiothoracic ratio, Cardiac magnetic resonance imaging, Chest radiograph, Cardiac imaging

## Abstract

**Background:**

Cardiothoracic ratio (CTR) in chest radiographs is still widely used to estimate cardiac size despite the advent of newer imaging techniques. We hypothesise that a universal CTR cut-off value of 50% is a poor indicator of cardiac enlargement. Our aim was to compare CTR with volumetric and functional parameters derived from cardiac magnetic resonance imaging (MRI).

**Methods:**

309 patients with a chest radiograph and cardiac MRI acquired within a month were reviewed to assess how CTR correlates with multiple cardiac MRI variables: bi-ventricular EDV (absolute and indexed to body surface area), EF, indexed total heart volume and bi-atrial areas. In addition, we have also determined CTR accuracy by creating multiple ROC curves with the described variables.

**Results:**

All cardiac MRI variables correlate weakly but statistically significantly with CTR. This weak correlation is explained by a substantial overlap of cardiac MRI parameters in patients with normal and increased CTR. For all variables, CTR was only mildly to moderately better than a chance to discriminate cardiac enlargement (AUC 0.6–0.7). Large CTR values (> 55%) are specific but not sensitive, while low CTR values (< 45%) are sensitive but not specific. Values in between are not sensitive nor specific.

**Conclusions:**

CTR correlates weakly with true chamber size assessed by gold standard cardiac MRI and has a weak discriminatory power. Thus, clinical decisions based on intermediate CTRs (45–55%) should be avoided. Large CTRs (> 55%) are likely indicative of true heart chamber enlargement. Low CTRs (< 45%) are likely indicative of normal heart size.

**Supplementary Information:**

The online version contains supplementary material available at 10.1186/s13244-021-01097-0.

## Key Points

- CXR-derived CTR correlates weakly with cardiac chamber enlargement detected on cardiac MRI.
- No single CTR cut-off value has good accuracy in diagnosing cardiomegaly.
- The cut-off value of CTR > 50% has limited sensitivity and specificity.
- Intermediate CTR values (45-55%) do not reliably indicate heart size.

## Background

Chest radiographs (CXR) are routinely used to assess the lungs and mediastinum. Cardiothoracic ratio (CTR) is a simple method to evaluate the heart size on chest radiographs. Despite having been introduced more than 100 years ago [1], it is still commonly reported nowadays, even though new imaging techniques have been developed. This is probably because CTR can be easily measured and simply interpreted on a widely available and inexpensive imaging study. Nevertheless, CTR depends on multiple technical and anatomic factors that can contribute to an inaccurate assessment of the real heart size. CTR is only considered reliable if calculated from a frontal upright postero-anterior (PA) chest radiograph. The supine position and any other antero-posterior (AP) projection overestimate cardiac size due to a magnification effect caused by the heart being closer to the imaging cassette. In addition, there are other cardiac and non-cardiac aspects such as sub-optimal inspiratory effort, patient’s or thoracic cage abnormalities that could influence the value of CTR [2–4].

CTR is defined as the ratio between the maximal horizontal cardiac diameter and the maximal horizontal inner thoracic cage diameter [2]. A CTR > 0.5 (or > 50%) is considered abnormal. In radiology reports, terms like “cardiomegaly” or “increased heart size” are commonly used to describe an increased CTR. Despite its broad use, some researchers have questioned the real value of this arbitrary cut-off point. For example, in a study with patients undergoing coronary angiography due to angina, a CTR between 42 and 49% was associated with a higher risk of all-cause mortality or major coronary event (death, non-fatal myocardial infarction) compared to patients with CTR < 42% [5].

In modern diagnostic imaging, newer modalities allow to thoroughly examine the three-dimensional structure of the heart, assessing chamber size and function. Some studies have questioned the correlation between CTR and cardiac parameters derived from other imaging techniques with controversial results. In comparisons done within specific cardiac pathologies, there is usually an absence of correlation with CTR or CTR correlates with the size of only one or two cardiac chambers [6, 7]. A systematic review by Loomba et al. showed that CTR is sensitive for identifying an increased left ventricular volume on echocardiography (sensitivity 83.3%) but lacks specificity (45.4%) [8]. Larger CTRs were related to increased mortality risk in adults with congenital heart disease [6]. Apart from chest radiographs, CTR can also be measured using other imaging modalities like CT and MRI. However, Schlett et al. found that CTR derived from CT scout images did not correlate to LV parameters measured from the same CT scan [9].

Cardiac MRI is a modern, high-resolution, ionising radiation-free technique that is now considered the gold standard for cardiac volumetry and function assessment [10–12]. We hypothesise that the cut off value of CTR = 50% is a poor indicator of cardiac enlargement and should be interpreted with caution. The primary aim of this study was to compare the CTR to indexed cardiac volumetry values. Secondary objectives were: (1) to evaluate the isolated correlation between CTR and individual cardiac chamber measurements, (2) to stratify the results by age and gender using the dedicated normal cardiac MRI values for comparison.

## Methods

### Study population

**Fig. 1:**
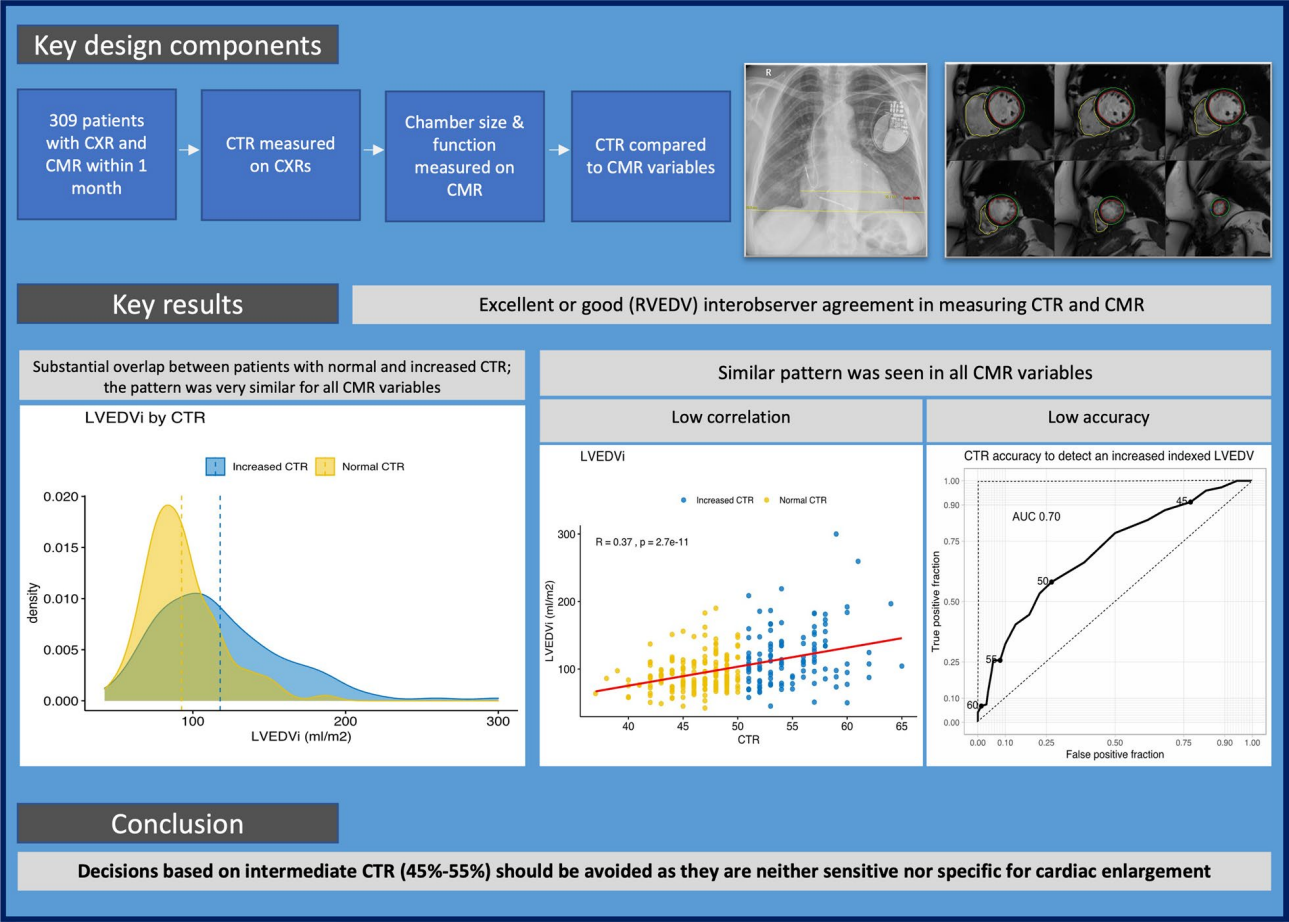
Key components of study design and results

Institutional Review Board approval was waived by our Research and Innovation Department, which classified the study as Service Evaluation. A retrospective single centre study was performed. A random list of 532 chest radiographs performed between 2016 and 2020 was selected with the following inclusion criteria: (1) adults (> 18 years old) with a chest radiograph and (2) cardiac MRI performed in a time period of less than one month. The initial list was reduced to 438 patients after excluding duplicate entries (patients with more than one radiograph) and excluding patients with AP chest radiographs. Only PA chest radiographs were considered for analysis. Additionally, cases with obscured heart contours due to pleural effusion or pulmonary consolidation were excluded. Regarding cardiac MRI, patients were excluded if the scan was incomplete (i.e. no ventricular functional module acquired) or if the scan quality was severely affected by artefacts resulting in non-analysable datasets. Once the exclusion criteria were applied, the final sample was formed by 309 patients. Figure 1 shows the key components of the study design.

### Chest radiographs

**Fig. 2:**
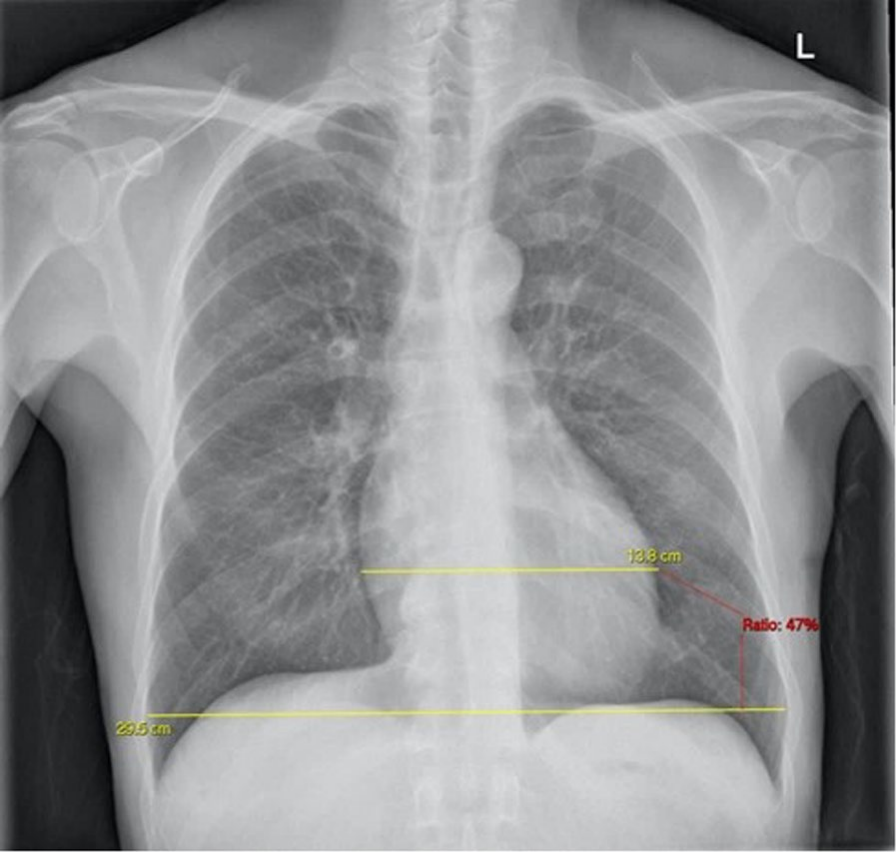
The cardiothoracic ratio on PA chest radiograph. The maximum transverse cardiac diameter is divided by the maximum transverse diameter of the thorax and multiplied by 100

Two radiologists (J.N., P.S.) measured the CTR dividing the largest horizontal heart diameter by the largest horizontal internal thoracic cage diameter (Fig. 2) using a DICOM viewer software package (Carestream VUE 12.2.2.2, Carestream Health Inc).

### Cardiac MRI

Studies were performed on 1.5-T and 3-T cardiac MRI systems (Aera and Vida, Siemens Medical Solutions; Achieva, Philips Healthcare) with breath-holding and electrocardiography (ECG)-triggering techniques. All analysed studies had a ventricular functional module available (horizontal long axis, vertical long axis and LVOT views, and a short axis stack from base to apex).

Cardiac MRI scans were analysed using dedicated post-processing software (CVI^42^ Version 5.11.4, Circle Cardiovascular Imaging Inc) by two radiologists with 2- and 3-years of experience in cardiac MRI analysis (PS, MG), with the senior review by a Level 3 certified cardiac MRI practitioner (M.A.) where necessary.

The following protocol was performed: firstly, automated left and right ventricular volume analyses were obtained. Then, the software traced biventricular endocardial and LV epicardial borders in end-diastole and end-systole to obtain the end-diastolic volumes (EDV), end-systolic volumes (ESV), bi-ventricular ejection fraction (EF) and LV mass. A manual correction was applied where needed. Papillary muscles were included in the blood pool when measuring ventricular volumes and excluded from the blood pool when calculating LV mass to increase the accuracy and align with the methods of the normal reference studies [15]. The end-systolic atrial areas and volumes were also obtained using horizontal and vertical long-axis cine images. All obtained values were indexed to the patient’s body surface area, calculated using the Mosteller formula. Additionally, the indexed total heart volume (THVi) was calculated by adding LVEDVi, RVEDVi, indexed LA volume and indexed RA volume.

After performing the measurements, studies were classified into nine different diagnostic categories: ischaemic heart disease, non-ischaemic cardiomyopathy, myocarditis/pericarditis, heart failure/LV dysfunction, valvulopathy, congenital heart disease, ≥ 2 pathologies, normal/screening and other diseases. The recorded diagnoses were obtained based on the original report and combined with the researchers’ observations. In cases of discrepancy between the researchers’ interpretation and the reported diagnosis, a consensus was reached by reviewing the studies in a team with a Level 3 certified cardiac MRI practitioner (M.A.).

### Interobserver variability

Interobserver variability was assessed by a second analysis of approximately 10% of the total sample: 30 CXR and 30 cardiac MRI studies were randomly selected.

### Data analysis

Statistical analysis was performed using SPSS (version 25.0). First, normality was evaluated using the Shapiro–Wilk test. According to the results, the following statistics were applied: Spearman correlation ranks to assess correlations, Mann–Whitney U test for comparison between groups and the Intraclass correlation coefficient (ICC) for interobserver variability (absolute agreement estimated using a two-way mixed effect model). Statistically significant differences were defined by a *p*-value less than 0.05. Graphics and Receiver operating characteristic (ROC) analysis were obtained using R and its library plotROC [13, 14].

Categorical data are expressed as counts and percentages. Continuous variables are presented as mean ± standard deviation or as median with inter-quartile range.

## Results

### Study population and baseline characteristics

Among 309 total individuals, 215 (69.6%) were males and 94 (30.4%) females. The average patient’s age was 57.9 ± 14.8 years. There was no significant age difference between males and females (58 ± 16 vs. 58 ± 15 years, respectively; *p* > 0.05). The average time interval between cardiac MRI and chest radiographs was 9.08 ± 4.95 days.

Cardiac MRI diagnoses and their distribution are summarised in Table 1. Ischaemic heart disease was the most common pathology, with 114 cases (36.9%). 25 (8.1%) studies were normal. Cases with diagnoses that were less common and could not be assigned to any of the pre-defined main groups were included in “Other”.

**Table 1:** Distribution of diagnoses and their characteristics

Pathology	Cases (%)	Normal: increased CTR	♀:♂
Ischaemic heart disease	114 (36.9)	77:37	25:89
Non-ischaemic cardiomyopathy	67 (21.7)	32:35	23:44
Myocarditis/pericarditis	22 (7.1)	14:8	7:15
Heart failure/LV dysfunction	12 (3.9)	9:3	4:8
Valvulopathy	22 (7.1)	13:9	12:10
Congenital heart disease	6 (1.9)	1:5	1:5
≥ 2 pathologies	21 (6.8)	14:7	4:17
Normal/screening	25 (8.1)	22:3	12:13
Other	20 (6.8)	12:8	6:14

### Reproducibility of CTR and cardiac MRI measurements

There was an excellent interobserver agreement in CTR measurements (ICC estimate 0.937; 95% CI 0.869–0.97). Interobserver agreement was also excellent in all the analysed cardiac MRI parameters (ICC > 0.9) except in the RVEDV, where it was good (ICC 0.846), Table 2.

**Table 2:** Interobserver agreement demonstrated as intraclass correlation coefficient (ICC) for measuring CTR and cardiac MRI parameters

Parameter	ICC between observers
CTR	0.937 (0.869–0.97)
LVEDV	0.985 (0.962–0.994)
LVESV	0.992 (0.942–0.997)
LVEF	0.977 (0.936–0.99)
LV mass	0.984 (0.965–0.993)
RVEDV	0.846 (0.675–0.927)
RVESV	0.975 (0.919–0.99)
RVEF	0.935 (0.856–0.97)
LA volume	0.977 (0.948–0.99)
LA area	0.959 (0.914–0.98)
RA volume	0.926 (0.845–0.965)
RA area	0.91 (0.811–0.957)

Values in parentheses are 95% confidence intervals

*ICC* intraclass correlation coefficient, *CTR* cardiothoracic ratio, *MRI* magnetic resonance imaging, *LVEDV* left ventricular end-diastolic volume, *LVESV* left ventricular end-systolic volume, *LVEF* left ventricular ejection fraction, *LV* left ventricle, *RVEDV* right ventricular end-diastolic volume, *RVESV* right ventricular end-systolic volume, *RVEF* right ventricular ejection fraction, *LA* left atrium, *RA* right atrium

### Correlation of CTR and cardiac MRI parameters

**Fig. 3:**
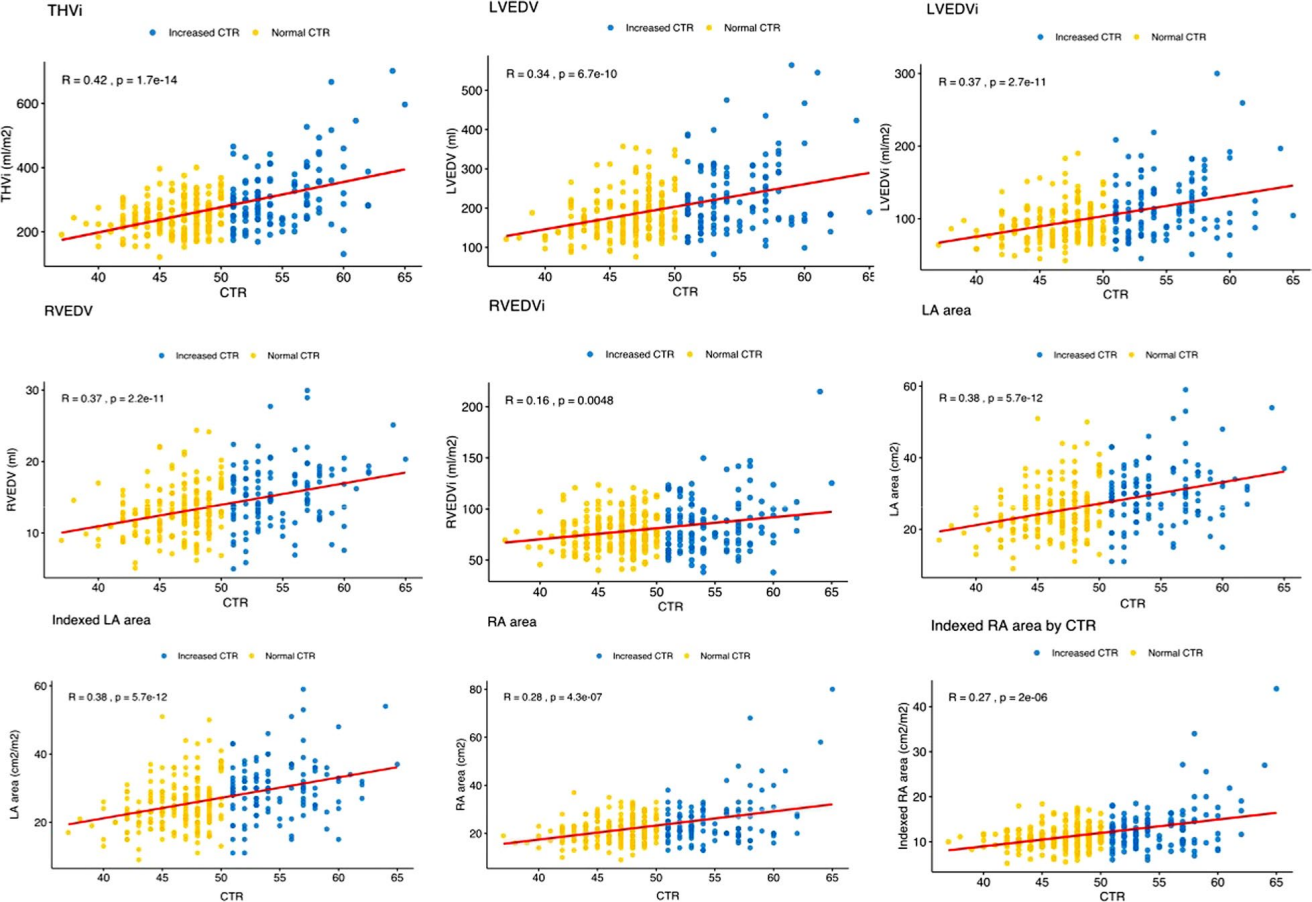
Scatterplots including correlation lines between CTR and cardiac volumes and areas. Yellow dots represent patients with CTR < 50%, blue dots patients with CTR > 50%. Although there is a weak trend for patients with larger CTR to have larger ventricular volumes and atrial areas, the majority of measured cardiac *MRI* parameters are noted in both individuals with normal and increased CTR. *THVi* total heart indexed volume, *LVEDV* left ventricular end-diastolic volume, *LVEDVi* left ventricular indexed end-diastolic volume, *RVEDV* right ventricular end-diastolic volume, *RVEDVi* right ventricular indexed end-diastolic volume, *LA* left atrium, *CTR* cardiothoracic ratio

THVi showed the maximum correlation with CTR values (r = 0.41). All other cardiac MRI parameters showed weaker correlations (Table 3, Fig. 3), which was nearly negligible in the case of RVEDVi. As expected, all parameters showed positive correlations except biventricular ejection fraction, which was negatively correlated with CTR. All correlations were statistically significant (*p* < 0.005).

**Table 3:** MRI parameters between normal vs increased CTR and CTR correlation with cardiac MRI parameters

MRI parameter	CTR ≤ 50 (N = 194)	CTR > 50 (N = 115)	CMR and CTR correlation
LVEDVi (ml/m^2^)	89 (76–106)	110 (86–139)	0.367 (*p* < 0.005)
LVEF (%)	54 (42.5–61)	42 (29–55)	− 0.341 (*p* < 0.005)
Indexed LV mass (mg/m^2^)	68 (60–78.5)	80 (66–98)	0.319 (*p* < 0.005)
RVEDVi (ml/m^2^)	75 (64–89)	84 (66–102)	0.170 (*p* < 0.005)
RVEF (%)	58 (53–63)	54 (41–59)	− 0.241 (*p* < 0.005)
Indexed LA volume (ml/m^2^)	40 (32–50)	56 (40–75)	0.389 (*p* < 0.005)
Indexed LA area (cm^2^/m^2^)	13 (10–15)	15 (12–18)	0.360 (*p* < 0.005)
Indexed RA volume (ml/m^2^)	33.5 (27–42.5)	40 (31–58)	0.247 (*p* < 0.005)
Indexed RA area (cm^2^/m^2^)	11 (9–12)	12 (10–15)	0.249 (*p* < 0.005)

Values in the two middle columns are median (interquartile range), and values in the far-right column are correlation coefficients

*MRI* magnetic resonance imaging, *CTR* cardiothoracic ratio, *LVEDVi* left ventricular indexed end-diastolic volume, *LVEF* left ventricular ejection fraction, *LV* left ventricle, *RVEDVi* right ventricular indexed end-diastolic volume, *RVEF* right ventricular ejection fraction, *LA* left atrium, *RA* right atrium

### Comparison of patients with normal and increased CTR

**Fig. 4:**
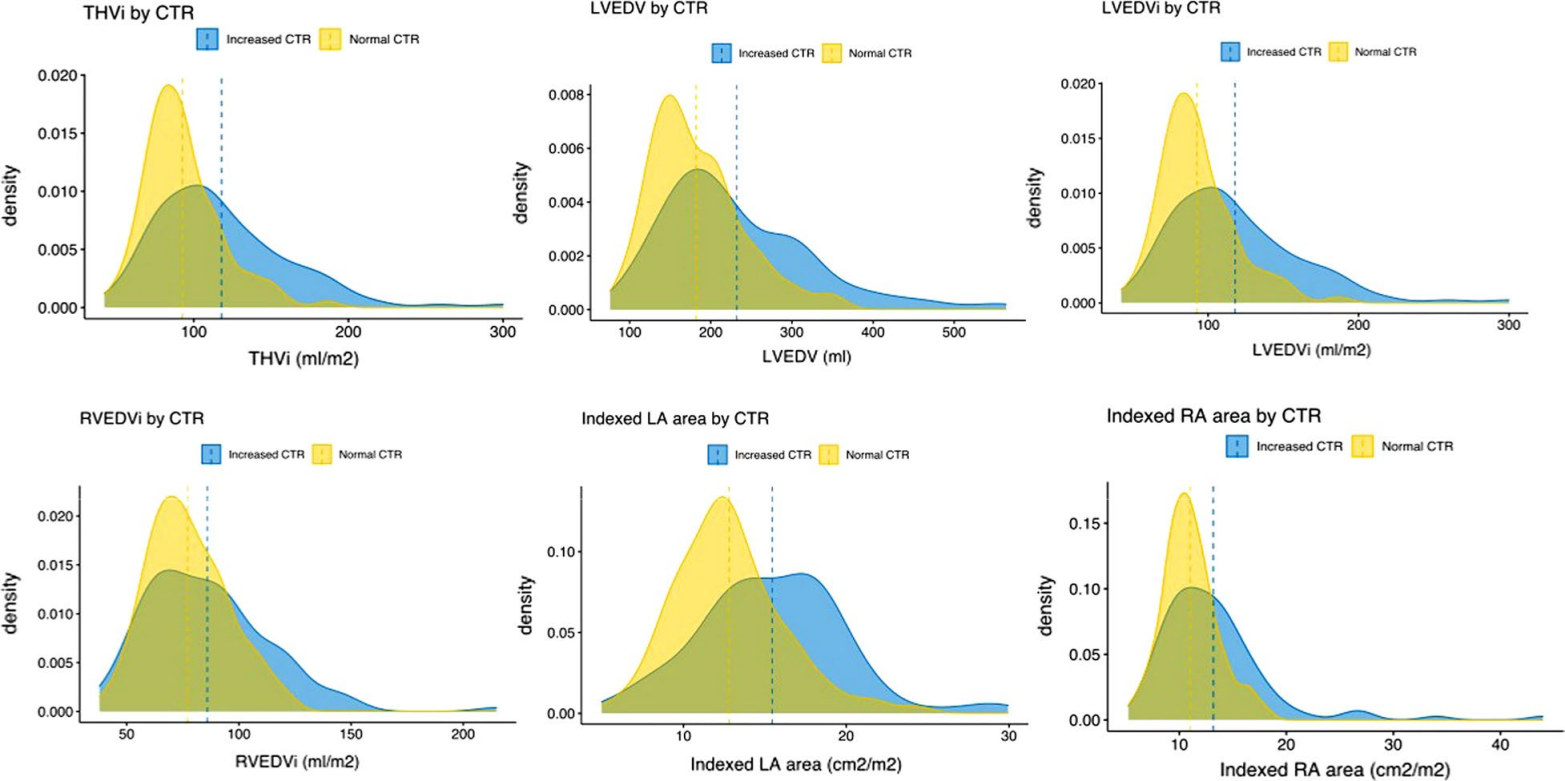
Density plots representing distributions of cardiac MRI parameters in patients with normal and increased CTR. Density plots are a variation of histograms that allow to observe the distribution of a variable in a dataset in a continuous fashion. They are instrumental in demonstrating the extensive overlap of cardiac MRI derived values between patients with normal and increased CTR. Dashed lines represent the means for each distribution. Note that mean values are larger in patients with CTR > 50%. This explains the described positive but weak correlations. *THVi* total heart indexed volume, *LVEDV* left ventricular end-diastolic volume, *LVEDVi* left ventricular indexed end-diastolic volume, *RVEDVi* right ventricular indexed end-diastolic volume, *LA* left atrium, *RA* right atrium, *CTR* cardiothoracic ratio

The CTR on CXR was normal in 194 cases (62.8%) and > 50% in 115 cases (37.2%). When grouped, patients with an increased CTR showed more abnormal cardiac MRI parameters than those with normal CTR (expressed as higher average/median ventricular volumes and atrial areas or lower ventricular ejection fraction) (Table 3). These differences were statistically significant. Nevertheless, there was a substantial overlap of individual cardiac MRI values between groups, which explains the low correlation coefficients (Figs. 3, 4).

**Fig. 5:**
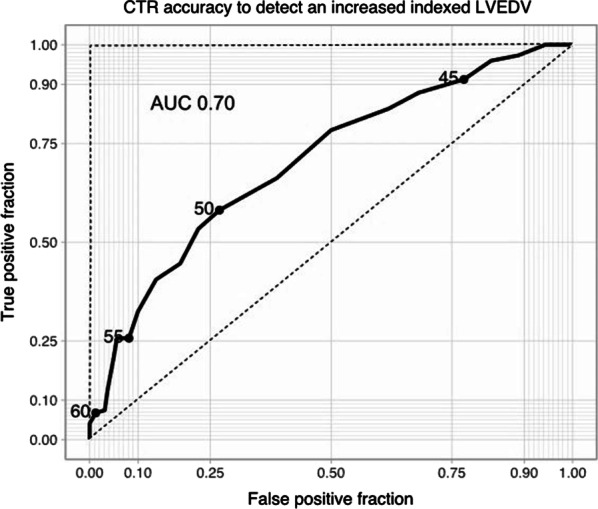
ROC curve of CTR and increased LVEDV. ROC curve illustrating diagnostic accuracy of multiple CTR cut-off values to detect an increased LVEDVi. The *X*-axis shows true positives for each cut-off (an increased LVEDV on cardiac MRI is seen by the defined cut-off). The *Y*-axis shows false positives (normal LVEDV on cardiac MRI, which is characterised as increased based on a given CTR value). Higher CTR values give few false positives at the expense of many false negatives (many patients with an increased LVEDV are missed). Lower CTR values diagnose most patients with an increased LVEDV at the expense of many false-positive results. The area under the ROC curve describes the overall diagnostic power of CTR. An ideal test would have no false positives or false negatives (AUC = 1 described by the 90° dashed line). Random guessing would render 50% of true positives and 50% of false positives (AUC = 0.5) and is represented by the no-discrimination line (45° dashed diagonal line). *CTR* cardiothoracic ratio, *LVEDVi* left ventricular indexed end-diastolic volume, *AUC* area under the curve. Other parameters are available in Additional file 1: Fig. S1

Biventricular cardiac MRI parameters were grouped into normal and abnormal according to their age and sex reference values [15]. There was a weak but significant association (*r*_φ_ < 0.4, *p* < 0.05) between an increased CTR, increased indexed biventricular volumes, increased LV mass and decreased biventricular ejection fraction. ROC curves were used to represent the sensitivity and specificity of multiple CTR values graphically. A truly increased heart size was defined as increased LVEDV, LVEDVi, RVEDVi, LA area, and RA area. True-positive results (Se) and false-positive results (1-Sp) were plotted together for each variable. The diagnostic accuracy of CTR was then inferred from the area under the curve (AUC). For all variables, CTR was only mildly to moderately better than the chance to discriminate cardiac enlargement (AUC 0.6–0.7) with a large number of false positives (low Sp) and false negatives (low Se), see Table 4. However, low CTR cut-off points produced very few false-negative results, and large CTR values have few false-positive results (Fig. 5, Additional file 1: Fig. S1).

**Table 4:** Diagnostic accuracy of CTR = 51% for detecting abnormal cardiac MRI parameters by Receiver operating characteristic analysis

MRI parameter	AUC	Sensitivity, %	Specificity, %	False-negative, %	False-positive, %
LVEDVi	0.704 [0.646–0.761]	53.4 (79/148)	77.6 (125/161)	46.6 (69/148)	22.4 (36/161)
LVEF	0.669 [0.607–0.731]	44.6 (91/204)	77.1 (81/105)	55.4 (113/204)	22.9 (24/105)
Indexed LVmass	0.665 [0.590–0.739]	57.1 (40/70)	68.6 (164/239)	42.9 (30/70)	31.4 (75/239)
RVEDVi	0.719 [0.640–0.798]	67.9 (36/53)	69.1 (177/256)	32.1 (17/53)	30.9 (79/256)
RVEF	0.657 [0.590–0.723]	52.7 (58/109)	71.4 (142/200)	47.3 (52/109)	28.6 (57/200)

Values in parentheses are the numbers for percentage calculation. Values in brackets are 95% confidence intervals. *MRI* magnetic resonance imaging, *AUC* area under the curve, *LVEDVi* left ventricular indexed end-diastolic volume, *LVEF* left ventricular ejection fraction, *LV* left ventricle, *RVEDVi* right ventricular indexed end-diastolic volume, *RVEF* right ventricular ejection fraction

## Discussion

This study aims to determine the reliability of CTR to predict cardiac enlargement by comparing the CTR values to cardiac parameters. Our results have shown a weak but statistically significant correlation between CTR and cardiac MRI defined parameters related to cardiac size. This weak correlation is explained by the substantial overlap of all measured cardiac MRI values in patients with normal and increased CTR. Although patients with CTR > 50% tend to have larger ventricular volumes and atrial areas, many patients with a normal CTR have abnormal cardiac MRI values. Interestingly, CTR correlated significantly with all ventricular and atrial measurements, despite having a broad spectrum of pathologies. In a heterogeneous adult population of our study, the cardiac chamber responsible for an increased CTR is variable, as is the number of enlarged chambers.

Compared to a paediatric population study performed by Grotenhuis et al. [2] (including congenital heart diseases, aortic regurgitation and HCM), our results show that the THVi correlated better with CTR in adults than in children (*r* = 0.41 vs. *r* = 0.27). However, correlation with isolated chambers’ enlargement was substantially better in children. In paediatric patients with Tetralogy of Fallot, the RVEDVi and CTR correlated weakly (*r* = 0.4), and in those with aortic regurgitation, the LVEDVi and CTR showed moderate correlation (*r* = 0.5). In this regard, a more homogenous sample is a plausible explanation for the stronger correlation between ventricular volumes and CTR, as seen in paediatric patients (i.e. enlargement of the RV is the expected cause of increased CTR in patients with Tetralogy of Fallot, as is dilated LV in patients with aortic regurgitation). On the other hand, Spiewak et al. [7] did not find a correlation between CTR and ventricular volumes in patients with Tetralogy of Fallot but reported a weak but statistically significant correlation with atrial volumes. This weak correlation is maintained in our adult population. Anyhow, the commented heterogeneity of results among studies is in keeping with the poor discriminatory value of CTR regarding true cardiac size, which is likely translatable in routine settings.

Notably, patients with several cardiac pathologies have been included in the present study, thus covering a broad spectrum of cardiac morphologies. Nevertheless, reliance on CTR is based on the fact that even isolated chamber enlargement impacts the whole cardiac silhouette. Typically, LV dilatation causes leftward, downward and backward rotation of the heart in a clockwise direction. However, it can also result in anticlockwise rotation, which is not expressed as increased CTR on chest radiographs [2, 16]. Furthermore, RV does not contribute to the left heart contour unless severely dilated as the sternum limits its expansion. Only if dilated enough, RV may rotate leftwards, push the left ventricle and increase the left heart border [7]. This may help to explain the particularly low correlation found between CTR and RV volumes. As CTR effectively represents the transverse diameter between the RA and LV (which form the right and left heart borders, respectively), it might be expected to reflect more accurately the enlargement of these chambers. However, neither RA area nor LV volume showed stronger correlations with CTR than other parameters.

A large number of cases with normal CTR revealed another interesting aspect of our study. A normal CTR was determined in 62.8% of patients, even though only 8.1% of individuals had normal cardiac MRI scans. Therefore, a CTR < 50% on chest radiographs does not seem to be a reliable indicator to exclude cardiac pathology. Dimopoulos et al. [6] also found that only 56.4% of adult congenital heart disease patients had an increased CTR on chest radiographs. In addition, some authors state that even a normal CTR could be related to poor clinical outcomes [5, 17]. Jun et al. [17] determined that a CTR greater than 42% was an independent predictor of major adverse cardiac events after percutaneous coronary intervention. Of note, in our population, such values were rare, as only 22 individuals out of 309 (7%) had a CTR of ≤ 42%.

ROC curve analysis corroborated the poor discriminatory value of CTR with AUCs only mildly above “chance” when using cardiac MRI values as the gold standard. No single CTR cut-off point was able to classify increased and normal chamber sizes accurately. Low CTR thresholds were associated with many false positives (patients with higher CTR on CXR but normal heart size on cardiac MRI). Larger CTR thresholds failed to identify most patients with abnormal cardiac MRI parameters, thus giving very few true positive results. Yet, CTR showed value when extreme measurements were found. That is to say, the likelihood of having an increased heart size is high when a CTR > 55% is found, as is the likelihood of having a normal heart size with a CTR < 45%.

Although cardiac MRI is considered the gold standard to assess cardiac volumes and function, it is not always accessible or even indicated. Other imaging modalities such as echocardiography and CT are also available for cardiac evaluation. Meta-analysis performed by Loomba et al*.* showed that CTR was sensitive but not specific to detect left ventricular dilatation determined by echocardiography: after assessing six studies with a total of 466 patients, the authors concluded that CTR had 83.3% sensitivity, 45.4% specificity, 43.5% positive predictive value and 82.7% negative predictive value. However, after one study with a paediatric population was excluded, specificity significantly decreased to 25.2% [8]. On the contrary, CTR in patients with NSTEMI showed lower sensitivity (40%) but higher specificity (91%) in detecting cardiomegaly compared to echocardiography, however in this study, cardiomegaly was described as an enlargement of the right or left ventricle [18]. Furthermore, Chana et al. found CTR to have a limited value in detecting left and/or right ventricular dysfunction (0.7 AUC, 73.9% sensitivity and 47.4% specificity) [19].

Regarding CT, CTR can be derived from the CT itself, using the scout images or axial slices. While this allows overcoming the limitation of a time gap that previous studies encountered when comparing two different imaging modalities, it is important to note that CT images are acquired in the supine position. Schlett et al. used anteroposterior scout images to calculate the CTR and found that CTR did not correlate with CT derived end-diastolic LV volume, mass or size (all *p* ≥ 0.27). On the other hand, all these mentioned LV parameters showed significant correlation with a simple axial LV area-based measurement, suggesting that on CT, other straightforward measurements may be applied instead of traditional CTR [9]. Regarding CTR as a single parameter, CTRs obtained by CXR and CT correlated (*r* = 0.802) in a study of cancer patients by Gollub et al. The authors also showed a limited ability of CT-derived CTR to recognise LV hypertrophy determined by echocardiography (AUC was 0.71) [20].

Generally, the complexity of the heart and extracardiac factors, as described in our study, undermines the value of a single cut-off of CTR. Thus, our results support the evidence that the reliability of CTR to predict cardiac size and functional status is limited.

## Limitations

One of the possible limitations of our study is the heterogeneous study population. However, this allowed to assess CTR in a spectrum of pathologies in different categories, likely translatable to broader practice. Secondly, the study was performed in a specialised cardiothoracic centre, meaning there was a low proportion of healthy individuals in the study (8%). Thirdly, our selected maximum interval between chest radiographs and cardiac MRI was one month, although the calculated average time difference was nine days. It is reasonable to assume that a time gap between studies in some cases might have been associated with clinical changes. However, in daily practice, chest radiograph and cardiac MRI are rarely performed on the same day (only 7% in our study sample). Therefore further studies assessing this may give some insight on whether the timeframe could have an influence. Thirdly, it would be interesting to evaluate separate cardiac chamber variations in the heart silhouette and whether this complements CTR to make it a more accurate measure. In this regard, artificial intelligence may play a role in automating and defining cardiac morphology on chest radiograph suggestive of underlying cardiac chamber abnormality. Furthermore, even though cardiac MRI is considered as the gold standard for cardiac volumetry and function, regrettably, the technique, while being increasingly used, is not yet available in every setting and is time-consuming [21]. Hence, CXR cannot be replaced by MRI, but CTR, as an estimate of true cardiac size, should be interpreted with caution and correlated with other available imaging modalities. Finally, CTR itself has limitations due to various cardiac and non-cardiac factors [3, 4]. To address this, we have constrained the impact of this limitation by excluding AP radiographs or patients with pleural or pericardial effusions obscuring the cardiac borders on chest radiographs.

## Conclusions

Although CTR has an excellent interobserver agreement, is reproducible and easily measurable, it has little accuracy to distinguish a normal from an increased heart size when used as a single threshold method (i.e. CTR > 50%). Due to the lack of sensitivity and specificity of intermediate CTR values (45–55%), it would be more accurate to report a possibility of cardiac chamber enlargement rather than stating true cardiomegaly.

## Supplementary Information

**Additional file 1: Fig. S1**. ROC curves of CTR and: increased LVEDV, RVEDV and LV mass, decreased LVEF and RVEF. CTR cardiothoracic ratio, AUC area under the curve, LVEDV left ventricular end-diastolic volume, RVEDV right ventricular end-diastolic volume, LVEF left ventricular ejection fraction, RVEF right ventricular ejection fraction.

## Data Availability

The datasets used and/or analysed during the current study are available from the corresponding author on reasonable request.

## Abbreviations

AP: Antero-posterior
CTR: Cardiothoracic ratio
CXR: Chest radiograph
EF: Ejection fraction
LA: Left atrium
LV: Left ventricle
LVEDV: Left ventricular end-diastolic volume
LVEDVi: Left ventricular indexed end-diastolic volume
LVESV: Left ventricular end-systolic volume
LVESVi: Left ventricular indexed end-systolic volume
LVOT: Left ventricular outflow tract
MRI: Magnetic resonance imaging
PA: Postero-anterior
RA: Right atrium
RV: Right ventricle
RVEDV: Right ventricular end-diastolic volume
RVEDVi: Right ventricular indexed end-diastolic volume
RVESV: Right ventricular end-systolic volume
RVESVi: Right ventricular indexed end-systolic volume
THVi: Total indexed heart volume
