# Supplementary figures and images for: Limitations of cardiothoracic ratio derived from chest radiographs to predict real heart size: comparison with magnetic resonance imaging
Source: Insights Imaging. 2021 Nov 3;12:158. doi: 10.1186/s13244-021-01097-0 (PMC8566609; doi:10.1186/s13244-021-01097-0)

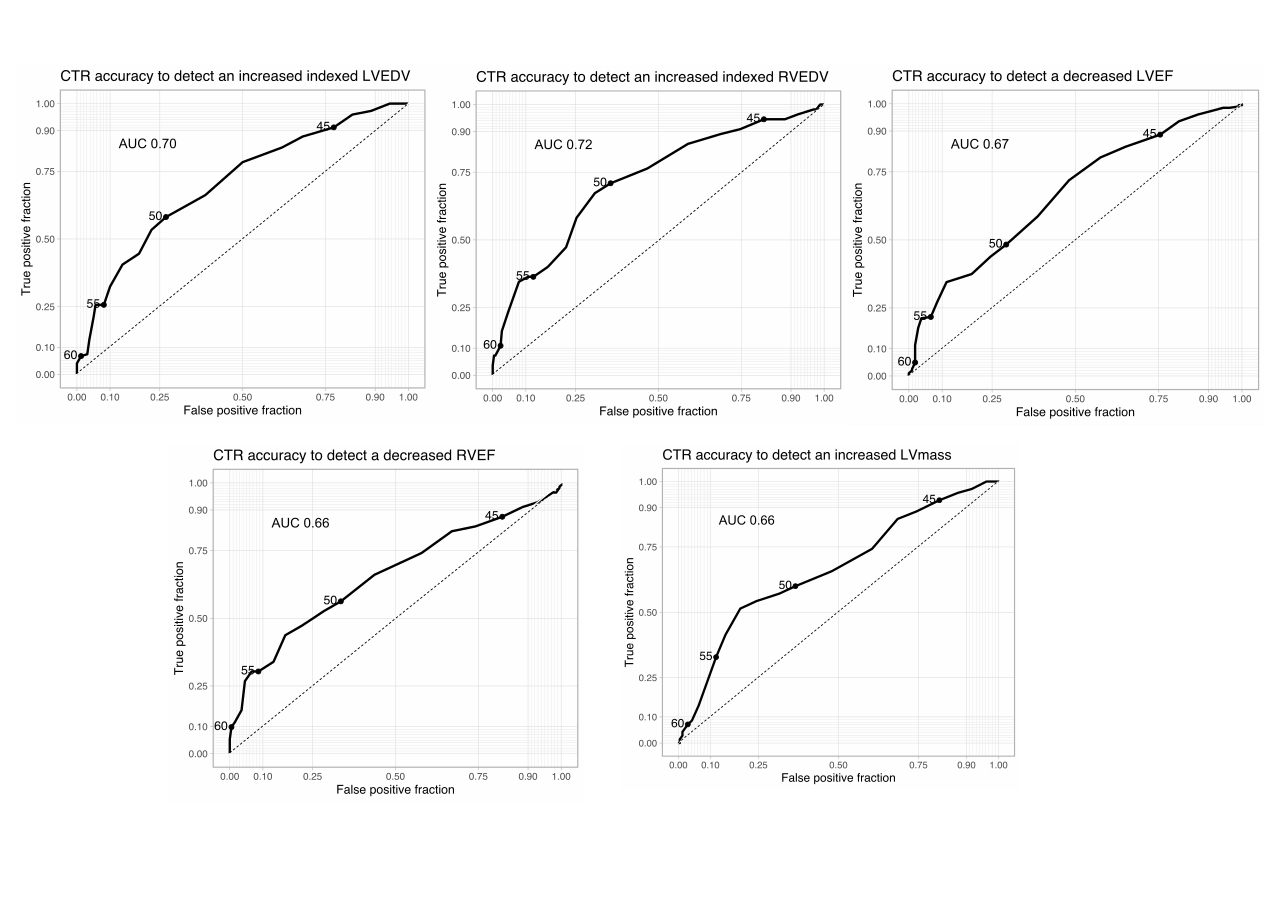

Supplement: Supplementary file 1 — Additional file 1: Fig. S1. ROC curves of CTR and: increased LVEDV, RVEDV and LV mass, decreased LVEF and RVEF. CTR cardiothoracic ratio, AUC area under the curve, LVEDV left ventricular end-diastolic volume, RVEDV right ventricular end-diastolic volume, LVEF left ventricular ejection fraction, RVEF right ventricular ejection fraction. [file 13244_2021_1097_MOESM1_ESM.jpg]
